# Photoreaction Pathways of Bacteriorhodopsin and Its D96N Mutant as Revealed by in Situ Photoirradiation Solid-State NMR

**DOI:** 10.3390/membranes12030279

**Published:** 2022-02-28

**Authors:** Arisu Shigeta, Yuto Otani, Ryota Miyasa, Yoshiteru Makino, Izuru Kawamura, Takashi Okitsu, Akimori Wada, Akira Naito

**Affiliations:** 1 Graduate School of Engineering, Yokohama National University, 79-5 Tokiwadai, Hodogaya-ku, Yokohama 240-8501, Japan; arisushigeta@gmail.com (A.S.); otani.yuto.dv@gmail.com (Y.O.); r.miyasa@gmail.com (R.M.); ymakino@med.kobe-u.ac.jp (Y.M.); 2 Laboratory of Organic Chemistry for Life Science, Kobe Pharmaceutical University, 4-19-1 Motoyamakitamachi, Higashinada-ku, Kobe 658-8558, Japan; okitsu@kobepharma-u.ac.jp (T.O.); w.akimori5139@gmail.com (A.W.)

**Keywords:** bacteriorhodopsin, light driven proton pump, photo-intermediate, photoirradiation solid-state NMR

## Abstract

Bacteriorhodopsin (BR) functions as a light-driven proton pump that transitions between different states during the photocycle, such as *all-trans* (AT; BR568) and 13-*cis*, 15-*syn* (CS; BR548) state and K, L, M_1_, M_2_, N, and O intermediates. In this study, we used in situ photoirradiation ^13^C solid-state NMR to observe a variety of photo-intermediates and photoreaction pathways in [20-^13^C]retinal-WT-BR and its mutant [20-^13^C, 14-^13^C]retinal-D96N-BR. In WT-BR, the CS state converted to the CS* intermediate under photoirradiation with green light at −20 °C and consequently converted to the AT state in the dark. The AT state converted to the N intermediate under irradiation with green light. In D96N-BR, the CS state was converted to the CS* intermediate at −30 °C and consequently converted to the AT state. Simultaneously, the AT state converted to the M and L intermediates under green light illumination at −30 °C and subsequently converted to the AT state in the dark. The M intermediate was directly excited to the AT state by UV light illumination. We demonstrated that short-lived photo-intermediates could be observed in a stationary state using in situ photoirradiation solid-state NMR spectroscopy for WT-BR and D96N-BR, enabling insight into the light-driven proton pump activity of BR.

## 1. Introduction

**Figure 1:**
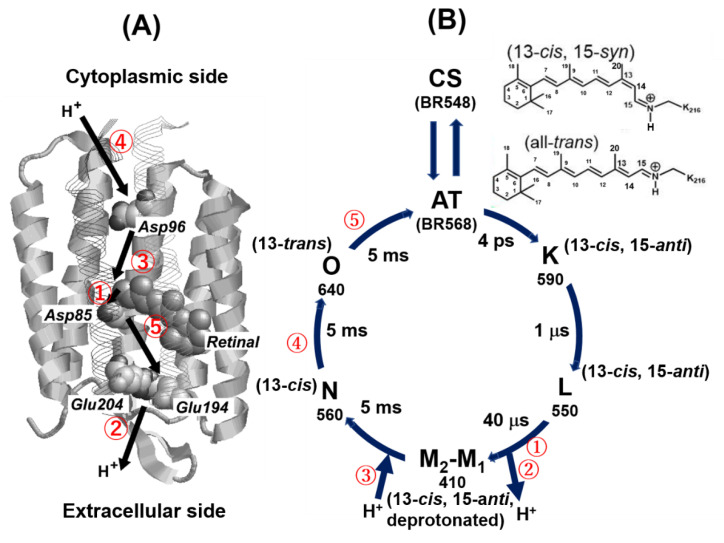
(**A**) Structure and proton transport pathways of bacteriorhodopsin (BR) (PDB: 1C3W). (**B**) Schematic of the BR photocycle. Approximate half-life for each transition and the absorption maximum of each intermediate are shown for the K, L, M_1_ (early M), M_2_ (late M), N, and O intermediates [7].

Bacteriorhodopsin (BR) is a retinal-containing transmembrane H^+^ pump found in the purple membrane (PM) of the archaeon *Halobacterium salinarum*, where it generates a proton gradient across the cytoplasmic membrane for ATP synthesis [1,2,3]. The 26 kDa BR protein contains seven transmembrane α-helices and a retinal group covalently bonded to the ε-amino group of Lys216 in helix G. In the extracellular region, an extensive three-dimensional hydrogen-bonded network of protein residues and seven water molecules connects the buried retinal Schiff base (SB) and the proton acceptor Asp85 to the membrane surface [4]. Energy from the light absorbed by the protein causes isomerization of the retinal from all-*trans* (AT; BR568) to 13-*cis*, 15-*anti* (CS; BR548) configurations to induce conformational changes in the protein, including a change in the tilt angle of the E and F helices. These structural changes suggest that protonation of Asp85 initiates a cascade of atomic displacements in the extracellular region, causing the release of a proton to the surface (see Figure 1A) [5,6].

Recently, a time-resolved serial femtosecond crystallographic structural study of BR using an X-ray-free electron laser revealed structural events that occur nanoseconds and milliseconds after photoactivation [7,8,9]. This time-resolved structural analysis revealed the sub-picosecond to millisecond retinal isomerization, proton release, proton uptake steps, and how the structural changes in BR achieve uni-directional membrane transport of H^+^ against a transmembrane concentration gradient.

The ordered sequence of conformational changes that occurs in BR as a result of light absorption is known as the photocycle (see Figure 1B) [7]. Upon illumination with green light (560 nm) at room temperature, the population of the AT state increases, a condition known as the light-adapted (LA) state. During the AT photocycle, the AT state is excited to the K-intermediate under green light illumination and is consequently relaxed through the L, M_1_ (early M) and M_2_ (late M), N, and O intermediates and ultimately returns to the AT (BR_568_) state (see Figure 1B).

During transition from the L to M intermediates, a H^+^ is transported from a SB to Asp85 via W402 (step ①), and a H^+^ is simultaneously released from a proton release group (PRG) such as Glu194 or Glu204 on the extracellular side (step ②). During transition from the M to N intermediate, a H^+^ is transported from Asp96 to a protonated Schiff base (SBH), which causes re-protonation of the SB (step ③). In the transition from the N to O intermediate, a H^+^ is transported from the cytoplasmic side to Asp96, which causes uptake of H^+^ from the cytoplasmic side to BR (step ④). In the last step of the photocycle, Asp85 re-protonates a PRG such as Glu194 or Glu204 in the transition from the O intermediate to the AT state (step ⑤). These stepwise changes in the sidechains induce a change in the pKa value (of up to 3 pH units) that results in H^+^ flux to the extracellular side of BR (see Figure 1A) [6,7,8,9,10,11].

In contrast to the AT photo-cycle that is related to proton pump activity, the photoreaction of the CS state (CS photocycle) has not been extensively studied. In previous studies [12,13,14,15], the CS state was irradiated with an Nd laser (530 nm) and an intermediate with a maximum absorbance of 610 nm was detected in the CS photocycle. As the same intermediate is not detected in the AT photocycle, it was thus determined to be an intermediate transformed from the CS state and assigned as the batho-13-*cis*-bR [13] or “610”-intermediate [12]. The intermediates are subsequently converted to the AT state through a leaking process from the CS photocycle. Therefore, one of the objectives in this study is to observe the photo-intermediates using in situ photoirradiation solid-state NMR of the CS photocycle with atomic resolution.

When the O intermediate transforms to the AT state in the photocycle of BR, a H^+^ is transported from Asp85 to a PRG through a hydrogen bond network consisting of Asp212 and Tyr185. As breakdown of the hydrogen bond network with the substitution of Tyr185 to Phe185 prevents H^+^ transport, it is thus predicted that the half-life of the O intermediate is significantly increased [16]. In a spectroscopic study of the Y185F-BR mutant, the dark-adapted (DA) state consisted of a CS:AT molar ratio of 3:1 [17,18]. Light-adaptation produces the AT state and the O intermediate [19]. Photoexcitation at −103 and −23 °C of the O intermediate produces an N intermediate, while the M intermediate is not found [20,21].

In the photoreaction cycle shown in Figure 1B, the photoreaction L ↔ M refers to proton exchange between SB and Asp85, while M ↔ N refers to proton exchange between Asp96 and SB. Therefore, replacement of Asp96 with Asn96 (D96N-BR) prevents the accumulation of any intermediate subsequent to the M intermediates, such as the N and O intermediates, while retaining proton pump activity [22,23,24,25]. Thus, the half-lives of the M and L intermediates are significantly increased in the D96N-BR mutant.

It is important to characterize the photo-intermediates to clarify the light-driven proton pump activity of BR. Photo-intermediates are normally detected based on the wavelength of the absorbance maximum using flash photolysis with pulsed laser light. For molecular characterization, solid-state NMR spectroscopy provides a powerful alternative for detecting intermediates during the photocycle. The L, M, and N intermediates have been detected by measuring ^13^C NMR signals of [^13^C]retinal-BR as well as ^15^N NMR signals of [ε-^15^N]Lys^216^-BR at a temperature range of 0 to −120 °C under illumination with light (λ > 540 for N and M, λ > 610 nm for L) [26,27]. The 13-*cis* form of [20-^13^C]retinal-BR can be distinguished from the 13-*trans* form, and the 15-*syn* form of [14-^13^C]retinal-BR can be distinguished from the 15-*anti* configurations based on the γ-position effects [28,29,30,31,32]. In another study, two protein structures corresponding to the AT and CS state retinal configurations were detected by observing rotational echo double resonance (REDOR) filtered ^13^C NMR signals of [1-^13^C]Tyr185-BR [33].

An in situ photoirradiation apparatus for the solid-state NMR spectrometer has been developed in which light passes through an optical fiber in the probe and the sample is irradiated from outside the rotor tube [31,34]. This system has been used to characterize an early M intermediate in the BR photocycle. The intermediate of the BR was trapped by irradiation of the LA state with light at >610 nm in a temperature range of −95 to −130 °C for at least 2 h [26,27]. In combination with the dynamic nuclear polarization (DNP) method, the heterogeneity of the dark-adapted BR and distortion in the K intermediates were revealed, and four distinctive L intermediates were detected [32,35]. In addition, photoirradiation DNP-enhanced solid-state NMR has been used to detect the photoreaction site of channelrhodopsin-2 combined with ^13^C double quantum filtering experiments [36].

Another type of in situ photoirradiation solid-state NMR apparatus was developed by our group, in which the sample is irradiated from inside the rotor tube through a glass rod inserted into the rotor [33,37,38,39,40,41,42,43,44,45]. This allows irradiation of the sample with extremely high efficiency and enables observation of the photo-intermediates as a stationary trapped state and the photoreaction processes of photoreceptor membrane proteins. In situ photoirradiation is particularly useful for the study of photo-cycles of retinal proteins such as sensory rhodopsin I (SR I) [38] and sensory rhodopsin II (SR II) [37,39], in which the late intermediates have long half-lives.

In this study, we focused on real-time detection of the photo-intermediate of WT-BR and D96N-BR using in situ photoirradiation solid-state NMR spectroscopy. Photoreaction pathways under photo-illumination with green and UV lights can be detected at low temperatures in such experiments. Notably, the photocycles of the CS and AT states were simultaneously investigated using in situ photoirradiation solid-state NMR experiments. Our aim is to gain insight into the structures and functions of the photo-intermediates using this methodology.

## 2. Materials and Methods

### 2.1. Materials

The *Halobacterium salinarum* retinal-deficient strain E1001 was grown in a temporary medium with the exogenous addition of [20-^13^C]retinal at a final concentration of 5 μM to yield selectively labeled [20-^13^C]retinal-WT-BR in the purple membrane (PM). Consequently, the PM with WT-BR was isolated according to a previously reported method [46].

The *H. salinarum* transformant for the D96N mutant, which was constructed using site-directed mutagenesis of bacterio-opsin, was used in this study. (The transformants were a gift by Dr. Satoru Tuzi). This *H. salinarum* (D96N-BR) strain was grown in a temporary medium containing [ε-^15^N]-Lys to yield selectively labeled [ε-^15^N]-Lys-D96N-BR in the PM. The PM was then isolated according to a previously reported method [46].

D96N bacterio-opsins (D96N-BOs) were prepared by photo-bleaching of the corresponding D96N-BR in hydroxylamine solution (500 mM, pH 7) at 4 °C under white light illumination for one day. Isotopically labeled regenerated [20-^13^C, 14-^13^C]retinal-D96N-BRs were obtained by the addition of [20-^13^C, 14-^13^C]retinal to the D96N-BOs obtained from bleached D96N-BR in a process termed in vitro retinal reconstitution [47]. Retinal bleaching and reconstitution were confirmed by measuring the absorbance of D96N-BR. 

All BR samples were then suspended in 5 mM *N*-(2-hydroxyethyl)piperazine-*N*’-2-ethan sulfonic acid (HEPES) buffer and 10 mM NaCl at pH 7. For NMR measurements, 5 mM Tris buffer with 300 mM NaCl at pH 9.0 was used.

### 2.2. Methods

#### 2.2.1. ^13^C and ^15^N Solid-State NMR Experiments

High-resolution solid-state ^13^C and ^15^N NMR spectra were recorded by cross polarization-magic angle spinning (CP-MAS) on a Chemagnetics CMX-400 Infinity FT-NMR spectrometer operated at 100.1 MHz for ^13^C, 40.3 MHz for ^15^N and 398.1 MHz for ^1^H. The spinning frequency was set at 4 kHz, and the probe temperature was kept at 20, −20, −30 and −60 °C using a temperature controller. The ^13^C chemical shifts were externally referenced to 176.03 ppm of glycine carbonyl carbon with respect to the tetramethylsilane (TMS) signal at 0 ppm. The ^15^N chemical shift value was referenced to 11.59 ppm of the amino nitrogen of glycine as an external reference that corresponds to ^15^NH_4_NO_3_ at 0 ppm.

#### 2.2.2. In Situ Photoirradiation Solid-State NMR Experiments

In situ photoirradiation was carried out using an optical fiber from outside the magnet through a tightly sealed cap made of a glass rod glued to a zirconia rotor (Figure S1). Photoirradiation was performed from the top of the spinner without touching the optical fiber to the glass cap of the rotor [37,38]. In this system, MAS operates under photo-illumination to generate high-resolution solid-state NMR signals. Thus, the MAS frequency was set at 4 kHz. The tip of the glass cap was inserted deeply and was ground to cast the light in random directions, including perpendicular to the glass rod. This allows the light to penetrate into samples that form a film on the inner wall from top to bottom of the rotor in which the absorbance is quite uniform and high (Figure S1). Using a CMX-400 Infinity NMR spectrometer equipped with this photoirradiation system, we were able to irradiate samples in the rotor with 100 mW green LED light (520 nm) and 50 mW UV LED light (365 nm) sources [37,38].

In the NMR measurements, a CP pulse sequence was used with a contact time of 1 ms, followed by acquisition with a 50 kHz TPPM proton decoupling pulse [48]. Typically, 20,000 transients were accumulated for the dark condition and light illumination with 520 or 365 nm LED light. 

#### 2.2.3. Observation of Photo-Intermediates and Photoreaction Pathways of BR

In the first step, the photoreaction pathways of the dark-adapted (DA) state which consists of AT and CS states of BR with a molar ration of ~ 1:1, were observed under dark conditions (D1) and subsequently observed under photo-illumination conditions (L1). In this experiment, the photoreaction pathways starting from the AT and CS states can be observed simultaneously. In the second step, the photo-reaction pathways of the light-adapted (LA) state, which consists of only the AT state, were observed, so the photoreaction pathway starting from the AT state alone can be clearly observed.

When BR is irradiated with green LED light, retinal absorbs the light energy and is excited from the AT state to the K intermediate and then subsequently relaxes to the L, M, N and O intermediates and finally returns to the AT state; this pathway is referred to as the photocycle (Figure 1B). It has been reported that the N intermediate exhibits a longer half-life in BR at −20 °C [27]. In this case, the N intermediate can be trapped in a stationary phase under continuous light illumination, as the light-activated production rate of the N intermediate is possibly faster than the relaxation decay rate. Thus, in a photocycle system, the photo-activation pathway of BR can be observed by accumulating the ^13^C CP-MAS NMR spectra initially under a dark condition (D1) and subsequently under irradiation with green LED light (L1) using the same acquisition parameters. The differences in spectra (L1-D1) between the dark (D1) and green light (L1) conditions provide a background-free spectrum, and photo-reacted 20-^13^C and 14-^13^C retinal signals distinctively appeared without protein and lipid signals, in which the reactant species exhibit negative peaks and the product species exhibit positive peaks. Thus, the signal assignments of photo intermediates and photoreaction pathways can be clearly characterized by inspecting the differences in spectra.

The retinal configuration of photo-intermediates can be determined by observing [20-^13^C]retinal-WT-BR and [20-^13^C, 14-^13^C]retinal-D96N-BR. Chemical shift values of [20-^13^C]retinal distinguish between 13-*trans* and 13-*cis,* and [14-^13^C]retinal distinguishes between 15-*anti* and 15-*syn* configurations.

## 3. Results and Discussion

### 3.1. Photoreaction Pathway of [20-^13^C]retinal-BR under Illumination with Green Light at 20 °C

**Figure 2:**
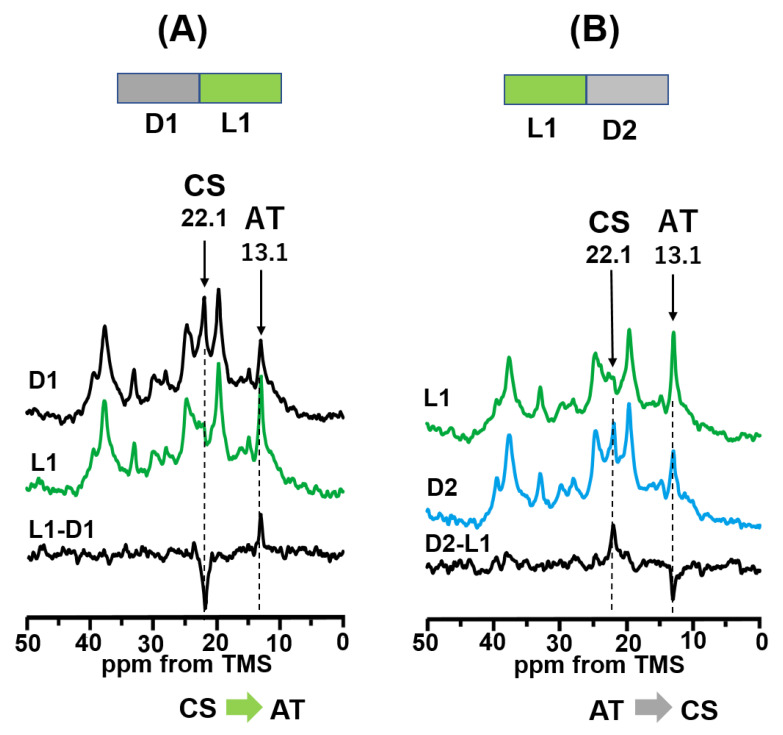
(**A**) Photo reaction pathway of [20-^13^C]retinal-WT-BR under green light (520 nm) illumination at 20 °C. ^13^C CP-MAS NMR spectra were recorded in the dark (D1; 20-^13^C) and under green light (L1; 20-^13^C), and the difference in spectra between L1 and D1 (L1-D1; 20-^13^C) indicated that the CS state changed to the AT state. (**B**) Photoreaction pathway in the dark after green light illumination at 20 °C. ^13^C CP-MAS spectra were recorded under green light (L1; 20-^13^C) and in the dark one day after L1 (D2; 20-^13^C), and the difference in spectra between D2 and L1 (D2-L1; 20-^13^C) indicated that the AT state changed to the CS state.

The DA state of BR was prepared by keeping the sample in the dark at 20 °C for one day. Afterward, ^13^C CP MAS NMR spectra of [20-^13^C]retinal-BR in the DA state were observed in the dark at 20 °C as shown in Figure 2A(D1). A CS signal appeared at 22.1 ppm and an AT signal appeared at 13.1 ppm (Table 1) with an intensity ratio of 1:1. When illuminating with green light, the CS signal decreased and the AT signal increased (Figure 2A(L1)). The difference in spectra between L1 and D1 is shown in Figure 2A(L1-D1). As mentioned in the Methods section, a negative peak represents reactant species (CS state) and a positive peak represents product species (AT state). Thus, the CS state converted completely to the AT state upon illumination with green light at 20 °C to produce an LA state (L1). After the sample was subsequently kept in the dark for one day, NMR signals were acquired to observe the DA state, as shown in Figure 2B(D2). Consequently, the difference in spectra between D2 and L1 was obtained, as shown in Figure 2B(D2-L1) which shows that one-half of the AT state changed to the CS state. In summary, only the CS state in the DA state changed to the AT state to produce the LA state under illumination with green light, and one-half of the AT state in the LA state changed to the CS state to produce the DA state under dark conditions.

### 3.2. Photoreaction Pathways of [20-^13^C]retinal-BR under Illumination with Green Light at −20 °C

**Figure 3:**
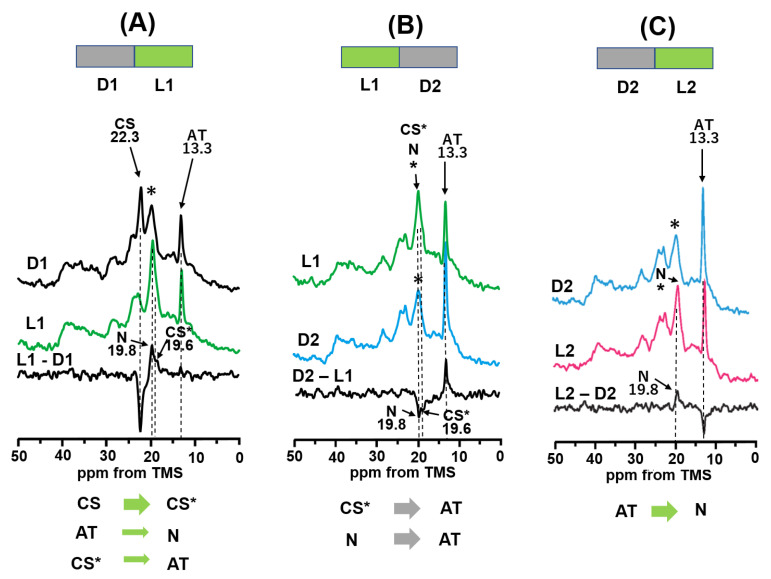
(**A**) Photo reaction pathway of [20-^13^C]retinal-WT-BR under green light (520 nm) illumination at −20 °C. ^13^C CP-MAS NMR spectra were recorded in the dark (D1; 20-^13^C) and subsequently under green light (L1; 20-^13^C), and the difference in spectra between L1 and D1 (L1-D1; 20-^13^C) indicated that the CS state changed to the CS* intermediate. (**B**) Photoreaction pathway in the dark after green light illumination at −20 °C. ^13^C CP-MAS NMR spectra were recorded under green light (L1; 20-^13^C) and subsequently in the dark after L1 (D2; 20-^13^C), and the difference in spectra between the D2 and L1 spectra (D2-L1; 20-^13^C) indicated that the CS* intermediate changed to the AT state. (**C**) Photoreaction pathway under green light illumination at −20 °C after the D2 state. ^13^C CP-MAS NMR spectra were recorded in the dark (D2; 20-^13^C) after L1 and subsequently under green light (L2; 20-^13^C), and the difference in spectra between L2 and D2 (L2-D2; 20-^13^C) indicated that the AT state changed to the N intermediate.

After the WT-BR was kept in the dark at 20 °C for one day to produce the DA state, the ^13^C CP-MAS NMR spectrum of [20-^13^C]retinal-WT-BR was observed in the dark at −20 °C as shown in Figure 3A(D1). CS and AT signals appeared at 22.3 and 13.3 ppm, respectively, in the dark-adapted (DA) state (Table 1). A signal (*) appearing at 20.0 ppm was assigned to the alkyl carbons of lipids in the PM. Subsequently, Figure 3A(L1) was recorded under illumination with green light at −20 °C to observe the photoreaction of WT-BR from the DA state. The CS state was significantly reduced, and * signals increased significantly. 

Figure 3A(L1-D1) shows the difference in spectra between L1 and D1 and indicates that the positive major signals of the CS* intermediate appeared at 19.6 ppm, and a positive minor signal of the N intermediate appeared at 19.8 ppm at −20 °C; these were not present at 20 °C. Specifically, the CS state is transformed to the CS* intermediate under green light illumination. The CS* intermediate forms a 13-*cis* configuration in reference to the chemical shift value of 20-^13^C as shown in Table 1. Thus, we demonstrated that the ^13^C NMR signal of the short-lived CS* intermediate could be observed in a stationary state at −20 °C using in situ photoirradiation solid-state NMR. Simultaneously, the N intermediate was transformed from the AT state during the AT photocycle as described later. 

After illumination with green light, signals were acquired in dark conditions (Figure 3B(D2)). We found that the CS* and N intermediates were reduced, and the AT state was increased. Thus, the difference in spectra between D2 and L1 (Figure 2B(D2-L1)) clearly shows that the CS* and N intermediates were converted to the AT state to produce an LA state (D2). 

This LA state was again illuminated with green light to observe the AT photocycle independently, as shown in Figure 3C(L2). The difference in spectra between L2 and D2 (Figure 3C(L2-D2)) indicates that a small fraction of the AT state converted to the N intermediate at 19.8 ppm. This result indicates that the AT photocycle exists, although only the N intermediate was trapped at −20 °C. However, the other intermediates (K, L, M and O) could not be detected at −20 °C, because their half-lives are too short to be detected using in situ photoirradiation solid-state NMR. It is thus noted that transition from the AT state to the N intermediate occurred in the process from D1 to L1. This result makes it clear that a positive peak at ~19.6 ppm (CS*) in Figure 3A(L1-D1) overlapped with the peak of the N intermediate at 19.8 ppm. In addition, the leakage process from the CS* to AT state coexisted under green light illumination. This resulted in an NMR peak for the AT state that apparently did not change, as shown in Figure 3A(L1-D1).

**Figure 4:**
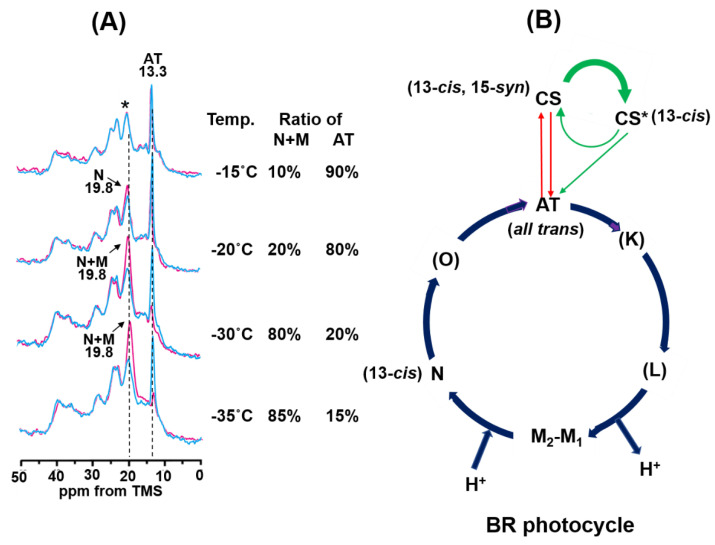
(**A**) ^13^C CP-MAS NMR spectra of [20-^13^C]retinal-BR under varying temperatures. Blue spectra were recorded in the dark for the LA state (retinal takes the AT configuration), and red spectra were recorded under green light illumination. The AT state changed to the N intermediate at −20 °C and to the N and M intermediates at −30 and −35 °C. (**B**) Photoreaction cycle of BR. In the AT photocycle, only the N-intermediate was detected. In the CS photocycle, the CS state was transferred to the CS* intermediate under green light illumination and consequently changed to the AT state upon dark exposure through a leakage process from the CS* intermediate to the AT state. (K), (L) and (O) intermediates were not detected using in situ photo-irradiation solid-state NMR.

In the CS photocycle, the CS state changed to the CS* intermediate under illumination with green light, and simultaneously the CS* intermediate partly changed to the AT state in the leakage process from the CS photocycle (Figure 4B). Subsequently, the CS* intermediate changed to the AT state in the dark to produce the LA state. In the AT photocycle, although the AT state changed to the K, L, M, N, and O intermediates and finally changed to the AT state at −20 °C, only the N intermediate was detected by in situ photoirradiation solid state NMR.

As shown in Figure 4A, in the AT photocycle, the trapped fraction of the N intermediate is significantly increased at a temperature lower than −20 °C. It has been reported that the M-intermediate significantly appears at a temperature lower than −20 °C and pH 10 and that 60% of the AT state converted to the M intermediate at −30 °C [27]. Considering this previous report, we revealed that the M intermediate significantly increased at 19.8 ppm in addition to the N intermediate at −30 °C. Furthermore, 85% of the AT state converted to the M and N intermediates at −35 °C. This may cause a slightly higher field shift of the (N+M+*) signal at −35 °C.

### 3.3. Photo-Reaction Cycle of [20-^13^C]retinal-BR

*In situ* photoirradiation solid-state NMR experiments demonstrated that unstable intermediates can be observed in a stationary state in real-time under photo-illumination. In the CS photocycle, the CS state is not directly converted to the AT state, but is converted through the CS* intermediate to the AT state under green light illumination, as shown in Figure 4B. The NMR signal of the CS* intermediate was observed for the first time in the CS photocycle of WT-BR using in situ photoirradiation solid-state NMR. At −20 °C, the CS* intermediate became a major component under green light illumination. As summarized in Table 1, the chemical shift value of the CS* intermediate is 19.6 ppm and that of CS is 22.3 ppm for (20-^13^C), thus the CS* intermediate has the same (13-*cis*) configuration as the CS state, although it differs slightly. In the active photocycle starting from the AT state (AT photocycle), photo-intermediates (K, L, M, N, O) could not be detected at 20 °C using in situ photoirradiation solid-state NMR because of their short half-lives. However, the N intermediate could be trapped at −20 °C, which is the only intermediate previously reported to be trapped at that temperature [26,27]. The fraction of the N+M intermediate significantly increased at a temperature lower than −20 °C, because the M intermediate starts appearing at below −20 °C and dominated at −30 °C. 

A summary of the photocycle starting from the DA state is given in Figure 4B. Upon photoirradiation of the DA state, the CS state transforms to the CS* intermediate in the CS photocycle under green light illumination, and the CS* intermediate partly transformed through a leakage process from the CS photocycle. Simultaneously, the AT state undergoes a photocycle through the K, L, M, N, and O intermediates; however, only the N and M intermediates could be observed using in situ photoirradiation NMR at a temperature lower than −20 °C. Next, we attempted to observe the M intermediate using D96N-BR, which has a longer half-life of the M intermediate than WT-BR.

### 3.4. Photo-Reaction Pathways as Revealed by [20-^13^C, 14-^13^C]retinal-D96N-BR under Green Light Illumination from the DA to LA State at −30 °C

**Figure 5:**
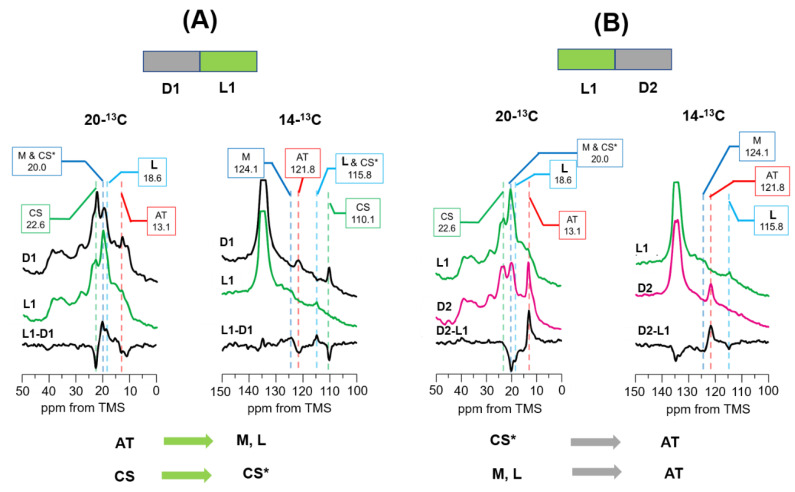
(**A**) Photoreaction pathways of [20-^13^C, 14-^13^C]retinal-D96N-BR under green light (520 nm) illumination at −30 °C. ^13^C CP-MAS NMR spectra were recorded in the dark (D1; 20-^13^C and 14-^13^C) and then under green light (L1; 20-^13^C and 14-^13^C), and the difference in spectra between L1 and D1 (L1-D1; 20-^13^C and 14-^13^C) indicated that the AT state changed to the M and L intermediates and the CS state changed to the CS* intermediate. (**B**) Photo reaction pathway of [20-^13^C, 14-^13^C]retinal-D96N-BR in the dark after green light illumination at −30 °C. ^13^C CP-MAS NMR spectra were recorded under green light (L1; 20-^13^C and 14-^13^C) and then in the dark (D2; 20-^13^C and 14-^13^C), and the difference in spectra between D2 and L1 (D2-L1; 20-^13^C and 14-^13^C) indicated that the CS* intermediate changed to the AT state and the M and L intermediates changed to the AT state.

The DA state of D96N-BR was prepared by keeping the sample in the dark at 20 °C for one day. Afterward, the ^13^C CP MAS NMR spectra of [20-^13^C, 14-^13^C]retinal-D96N-BR in the DA state were observed in the dark at −30 °C as shown in Figure 5A(D1). In the D1 spectrum, AT and CS signals of [20-^13^C and 14-^13^C] retinal appeared at (13.1 and 121.8 ppm) and (22.6 and 110.1 ppm), respectively. Figure 5A(L1) shows the ^13^C CP-MAS NMR spectrum of [20-^13^C, 14-^13^C]retinal-D96N-BR under illumination with green light (L1). In the L1 spectrum, the AT and CS signals were significantly reduced, and the signals around 20 ppm were increased.

The difference in spectra between L1 and D1 (Figure 5A(L1-D1)) indicated that the CS and AT peaks are negative, and the CS* (20.0 ppm; 20-^13^C, 115.8 ppm; 14-^13^C), M (20.0 ppm, 124.1 ppm) and L (18.6, 115.8 ppm) intermediate peaks are positive. The M intermediate was assigned by comparing the ^13^C chemical shift value of 20-^13^C and 14-^13^C with those previously reported for the M-intermediate [31,37] (Table S1). It has been reported that an N intermediate is not observed in D96N-BR, because the proton donor D96 is replaced by N96 [22,23,24,25]. Therefore, another peak was assigned to the L intermediate and the chemical shifts agree with previously reported values [31] (Table S1). These results clearly indicate that the CS state is mostly converted to the CS* intermediate under green light illumination at −30 °C. At the same time, the AT state is converted to the L and M intermediates. Notably, the L, M, and CS* intermediates with short half-lives were observed in real time as stationary states using in situ photoirradiation solid-state NMR. It is thus revealed that the CS state is not directly converted to the AT state, but is converted through the CS* intermediate similar to the case of WT-BR at −20 °C (as described in Section 3.2). 

After green light illumination, the NMR signals were observed in subsequent dark condition (Figure 5B(D2)) which may produce a LA state. The difference in spectra between D2 and L1 is shown in Figure 5B(D2-L1). The spectra clearly indicate that all CS* intermediate were converted to the AT state, and, L and M intermediates were converted to the AT state to form the LA condition, which consists entirely of the AT state. 

### 3.5. Photoreaction Pathways as Revealed by [14-^13^C, 20-^13^C]retinal-D96N-BR under Green Light Illumination from LA (D2) to DA (D3) States

**Figure 6:**
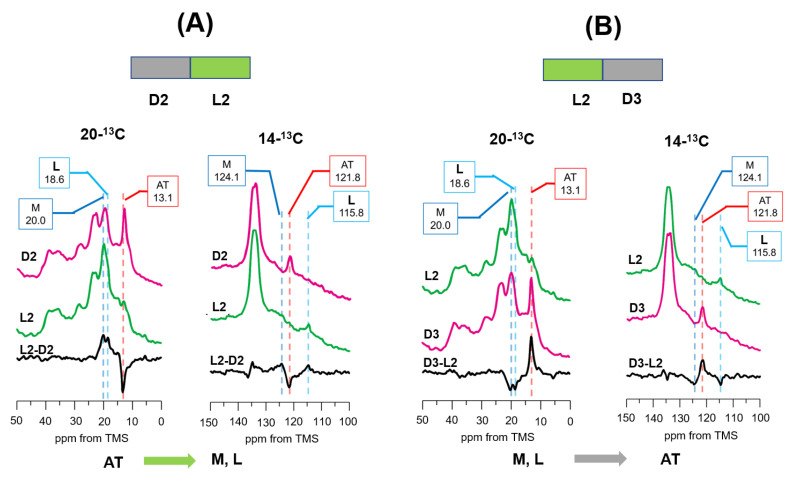
(**A**) Photo reaction pathways of [20-^13^C, 14-^13^C]retinal-D96N-BR under green light illumination from the light-adapted D2 condition at −30 °C. ^13^C CP-MAS NMR spectra were recorded in the dark (D2; 20-^13^C and 14-^13^C) and then green light (L2; 20-^13^C and 14-^13^C), and the difference in spectra between L2 and D2 (L2-D2; 20-^13^C and 14-^13^C) indicated that the AT state changed to the M and L intermediates. (**B**) Photoreaction pathways of [20-^13^C, 14-^13^C]retinal-D96N-BR in the dark from the L2 condition at −30 °C. ^13^C CP NMR spectra were recorded under green light (L2; 20-^13^C and 14-^13^C) and then in the dark (D3; 20-^13^C and 14-^13^C), and the difference in spectra between D3 and L2 (D3-L2; 20-^13^C and 14-^13^C) indicated that the M and L intermediates changed to the AT state.

Figure 6A (D2) shows the ^13^C CP-MAS NMR spectrum of the LA state, in which all retinal assumes the AT state. After green light illumination, the AT state was reduced and the L and M intermediates were increased (L2). Figure 6A(L2-D2) shows the difference in spectra between L2 and D2 for [20-^13^C, 14-^13^C] retinal-D96N-BR at −30 °C. It can be clearly seen that the AT state is transformed to the L and M intermediates during the AT photocycle. 

Figure 6B(L2) shows the ^13^C CP-MAS NMR signal of [20-^13^C, 14-^13^C]retinal-D96N-BR at −30 °C under green light illumination as the LA state. Following green light illumination, NMR signals were recorded in the dark (D3). Figure 6B(D3-L2) shows the difference in spectra between D3 and L2. The results revealed that the L and M intermediates were reduced, and the AT state was increased. This shows that the L and M intermediates are transformed to the AT state. As the CS state does not exist in the LA state, the CS* intermediate did not appear in this process.

### 3.6. Photoreaction Pathways as Revealed by [20-^13^C, 14-^13^C]retinal-D96N-BR under UV Light Illumination following Green Light Illumination

**Figure 7:**
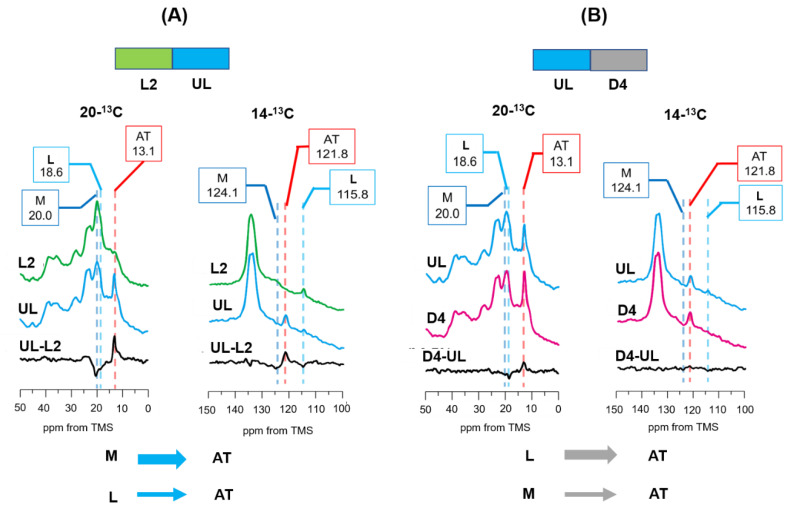
(**A**) Photoreaction pathway of [20-^13^C, 14-^13^C]retinal-D96N-BR under UV light (365 nm) illumination after green light illumination at −30 °C. ^13^C CP-MAS NMR spectra were recorded under green light (L2; 20-^13^C and 14-^13^C) and then under UV light (UL; 20-^13^C and 14-^13^C), and the difference in spectra between UL and L2 (UL-L2) indicated that the M-intermediate changed more significantly to the AT state compared to the L intermediate. (**B**) Photoreaction pathway of [20-^13^C, 14-^13^C]retinal-D96N-BR in the dark after UV light illumination at −30 °C. ^13^C CP-MAS NMR spectra were recorded under UV light (UL; 20-^13^C and 14-^13^C) and then in the dark (D4; 20-^13^C and 14-^13^C), and the difference in spectra between D4 and UL (D4-UL; 20-^13^C and 14-^13^C) indicated that the L intermediates changed to the AT state, and a small amount of the M-intermediate also changed to the AT state.

After green light illumination, the L2 spectrum was recorded and found to mainly contain the L and M intermediates, as shown in Figure 7A(L2). Subsequently, the L2 state under green light illumination was changed to illumination with UV light, as shown in Figure 7A(UL). The difference in spectra between UL and L2 is shown in Figure 7A(UL-L2). The L and M intermediates decreased and the AT state increased. The spectral difference between UL and L2 for [20-^13^C, 14-^13^C]retinal-D96N-BR indicated that the M intermediate was reduced more significantly than the L intermediate; this is more clearly seen in the spectrum for [14-^13^C]retinal-D96N-BR (Figure 7A(UL-L2)), where the L intermediate did not completely disappear. Consequently, following UV light illumination, the spectrum in the dark (D4) was acquired, as shown in Figure 7B(D4). Figure 7B(D4-UL) shows the difference in spectra between D4 and UL, which indicated that the remaining L intermediate transformed to the AT state and the M intermediate did not remain. This result indicates that there is a direct pathway from the M intermediate to the AT state by UV light illumination.

### 3.7. Photo-Reaction Pathways as Revealed by [ε-^15^N]Lys^216^-D96N-BR

**Figure 8:**
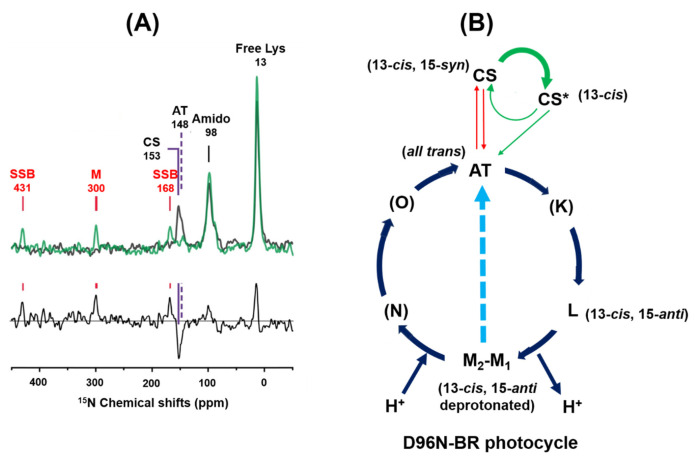
(**A**) ^15^N CP-MAS NMR spectra of [ε-^15^N]Lys-D96N-BR were recorded at −60 °C in the dark (black line) and under green light illumination (green line), and the difference in spectra between the black line and green lines was obtained as shown in the bottom line. The difference spectrum of [ε-^15^N]Lys^216^-D96N-BR indicated that the AT state changed to the M intermediate. (**B**) Photoreaction cycle of D96N-BR. In the AT photocycle, the L and M intermediates were detected, and the M intermediate directly changed to the AT state. In the CS photocycle, the CS state was changed to the CS* intermediate and then to the AT intermediate. (K), (N) and (O) intermediates were not detected using in situ photoirradiation solid-state NMR.

Figure 8A shows the ^15^N CP-MAS NMR spectra of [ε-^15^N]Lys-D96N-BR in the dark state (black line) and under illumination with green light (green line) at −60 °C. In the black line spectrum, the AT state appeared at 148 ppm and the CS state appeared at 153 ppm, which were assigned according to the previously reported ^15^N chemical shift values of [ε-^15^N]-Lys^216^-WT-BR^30^ (Figure 8A black line and Table S1). After illumination with green light, the protonated [ε-^15^N]-Lys^216^ NMR signal of the AT and CS states decreased and the signal of the deprotonated [ε-^15^N]-Lys^216^ at 300 ppm increased, which was assigned to the M intermediate. The difference in the spectra between the green and black lines (Figure 8A(bottom)) shows that the AT state (negative peak) converted to the M intermediate (positive peak). As the signal of the M intermediate appeared at 150 ppm lower field than those of the CS and AT states which form an protonated SB with [ε-^15^N]-Lys^216^, the M intermediate forms the deprotonated SB bond with [ε-^15^N]Lys^216^, which is in agreement with the previously reported chemical shift values of [ε-^15^N]Lys^216^ in a photo-excited M intermediate under green light illumination [30] (Table S1).

### 3.8. Photo Reaction Pathways in the D96N-BR Mutant

When DA (D1) D96N-BR was illuminated with green light, the CS state transformed to the CS* intermediate, which was stable at −30 °C and gradually transformed to the AT state through a leakage pathway from the CS* intermediate to the AT state. Therefore, the CS state is not directly transformed to the AT state, but passes through the CS* intermediate and subsequently changes to the AT state. Simultaneously, the AT state changed to the L intermediate then subsequently changed to the M intermediate during the AT photocycle. The M intermediate did not change to the N intermediate, instead changing to the AT state. The M intermediate is further directly excited to the AT state by UV light illumination (Figure 8B). A previous report showed that the back reaction from the M intermediate to the AT state under UV light illumination is significant for WT-BR, though not for D96N-BR [49]. Further work is therefore needed to clarify the existence of a photo reaction pathway from the M intermediate to the AT state under UV light illumination for D96N-BR.

Chemical shift values of the CS* intermediate appeared at 20.0 and 115.8 ppm for 20-^13^C and 14-^13^C, respectively. By comparing the chemical shift values of CS (22.9 ppm, 110.1 ppm), the CS* retinal configuration can be determined to be the 13-*cis* retinal configuration as shown in Table 1. Chemical shift values of the M intermediate were determined to be 20.0 ppm for 20-^13^C and 124.1 ppm for 14-^13^C, and it was assigned to the 13-*cis*, 15-*anti* retinal configuration. Chemical shift values of the L intermediate were determined to be 18.6 ppm for 20-^13^C and 115.6 ppm for 14-^13^C and it was assigned to the 13-*cis* retinal configuration, which agrees well with the previously reported chemical shift values for the L intermediate (Table S1) [31].

## 4. Conclusions

The ^13^C CP/MAS NMR signals of [20-^13^C]retinal-BR and [20-^13^C, 14-^13^C]retinal-D96N-BR were measured under green light illumination. The photochemical reaction pathway for [20-^13^C]retinal-BR was investigated by illuminating with green light at 20 °C. We clearly observed that the CS state was completely converted to the AT state to produce the LA state. Following exposure to a dark state for one day, half of the AT state converted to the CS state to produce the DA state. In the next step, D1(DA) was illuminated with green light at −20 °C. We observed that the CS state was converted to the CS* intermediate. This result indicates that the CS state is not directly converted to the AT state by green light illumination at −20 °C, but is converted to the AT state via the CS* intermediate. After green light exposure, the ^13^C CP-MAS NMR spectra were acquired in the dark. In this photoreaction pathway, the CS* intermediate was converted to the AT state, which produced an LA state. This LA state was illuminated with green light at −20 °C, and the AT state partly converted to the N intermediate through the AT photo cycle; the M intermediate significantly increased at temperatures lower than –20 °C Figure 4B summarizes the WT-BR photocycle obtained in this work.

Photoreaction pathways were observed for [20-^13^C, 14-^13^C]retinal-D96N-BR by green light illumination at −30 °C. We observed that the CS state was converted to the CS* intermediate and then changed to the AT state in the dark, and the AT state converted to the M and L intermediates, because the half-lives of the M and L intermediates are significantly increased in D96N-BR. The M and L intermediates were converted to the AT state in the dark to produce the LA state. This LA state (full AT state) was converted to the M and L intermediates, but not the CS* intermediate under green light exposure because the CS state does not exist in the LA state. We demonstrated that the unstable CS*, L and M intermediates could be observed in a steady state under illumination with green light at −30 °C. Also, the M intermediate was directly excited to the AT state by UV light. Thus, a photo-reaction pathway exists from the M intermediate to the AT state under illumination with UV light. The photocycle of D96N-BR obtained in this work is summarized in Figure 8B.

The present study demonstrates that in situ photoirradiation solid-state NMR is a powerful means to detect NMR signals in short-lived photo-intermediates in the DA and LA states of WT-BR and D96N-BR. In the CS photocycle, NMR signals of the CS* intermediate were stationarily detected in WT-BR and D96N-BR for the first time. In the AT photocycle, N and M intermediates were stationarily detected in WT-BR at −30 °C, and the L and M intermediates were stationarily detected in D96N-BR at −30 °C. Thus, not only the retinal structure but also the protein structures of photo-intermediates can be determined using in situ photoirradiation solid-state NMR spectroscopy.

## Figures and Tables

**Table 1:** ^13^C and ^15^N chemical shift values (ppm) of individual states of [20-^13^C, 14-^13^C]-retinal and [ε-^15^N]Lys-bacteriorhodopsin(WT-BR) and D96N-BR.

Protein	State	Temp.	20-^13^C	14-^13^C	[ε-^15^N]Lys^216^	Configuration
WT-BR	AT(BR568)	20 °C	13.1			all-*trans*
		−20 °C	13.3			all-*trans*
	CS(BR548)	20 °C	22.1			13-*cis*
		−20 °C	22.3			13-*cis*
	CS*	−20 °C	19.6			13-*cis*
	N	−20 °C	19.8			13-*cis*
D96N	AT(BR568)	−30 °C	13.1	121.8		all-*trans*
		−60 °C			148	all-*trans*
	CS(BR548)	−30 °C	22.6	110.1		13-*cis*, 15-*syn*
		−60 °C			153	13-cis, 15-syn
	CS*	−30 °C	20.0	115.8		13-*cis*
	M	−30 °C	20.0	124.1		13-*cis*, 15-*anti*
		−60 °C			300	13-cis, 15-*anti*
	L	−30 °C	18.6	115.8		13-*cis,*

CS*: State of intermediate that is transformed from CS state under green light illumination.

## Data Availability

All data generated in this study are available within this manuscript and Supplementary Materials.

## Supplementary Materials

The following is available online at https://www.mdpi.com/article/10.3390/membranes12030279/s1. Table S1. ^13^C and ^15^N chemical shift values (ppm) of individual states of [20-^13^C, 14-^13^C]-retinal and [ε-^15^N]Lys-bacteriorhodopsin(BR) and Y185F-BR reported in the literature. Figure S1. Schematic diagram of the in situ photoirradiation solid state NMR spectrometer. Figure S2. Full scale ^13^C CP-MAS NMR spectra of [Tyr^13^CO] and [20-^13^C, 14-^13^C]retinal-D96N-BR were recorded in the dark (D1; dark-adapted state) and then under green light (L1) illumination at −30 °C. Figure S3. Full scale ^13^C CP-MAS NMR spectra of [Tyr^13^CO] and [20-^13^C, 14-^13^C]retinal-D96N-BR were recorded in the dark (D2; light-adapted state) and then under green light (L2) illumination at −30 °C.
