# Supplementary material for: Photoreaction Pathways of Bacteriorhodopsin and Its D96N Mutant as Revealed by in Situ Photoirradiation Solid-State NMR
Source: Membranes (Basel). 2022 Feb 28;12(3):279. doi: 10.3390/membranes12030279 (PMC8949607; doi:10.3390/membranes12030279)
Supplement: Supplementary file 1 [file membranes-12-00279-s001.zip › membranes-1552277-supplementary.pdf]

Supplementary Materials

Photoreaction Pathways of Bacteriorhodopsin and Its D96N Mutant as Revealed by in Situ Photoirradiation Solid-State NMR

Arisu Shigeta [1], Yuto Otani [1], Ryota Miyasa [1], Yoshiteru Makino [1], Izuru Kawamura [1,*], Takashi Okitsu [2], Akimori Wada [2] and Akira Naito [1,*]

1 Graduate School of Engineering, Yokohama National University, 79-5 Tokiwadai, Hodogaya-ku, Yokohama 240-8501, Japan; arisushigeta@gmail.com (A.S.); otani.yuto.dv@gmail.com (Y.O.); r.miyasa@gmail.com (R.M.); ymakino@med.kobe-u.ac.jp (Y.M.);

2. Laboratory of Organic Chemistry for Life Science, Kobe Phamaceutical University, 4-19-1 Motoyamakitamachi, Higashinada-ku, Kobe 658-8558, Japan; okitsu@kobepharma-u.ac.jp (T.O.); w.akimori5139@gmail.com (A.W.)

* Correspondence: authors: kawamura-izuru-wx@ynu.ac.jp (I.K.), naito@ynu.ac.jp (A.N.)

Table S1. ^{13}C and ^{15}N chemical shift values (ppm) of individual states of [20-^{13}C, 14-^{13}C]-retinal and [ε-^{15}N]Lys^{216}-bacteriorhodopsin(BR) and Y185F-BR reported in the literature.

Protein	State	20-^{13}C	14-^{13}C	[ε-^{15}N]Lys^{216}	Configuration	Reference
BR	AT(BR568)	13.3[a]	122.0[a]	143.5[a]	all-*trans*<br>(13-*trans*, 15-*anti*)	a
	CS(BR548)	22.0[a]	110.5[a]	150.6[a]	13-*cis*, 15-*syn*	a
	K		118.2[d]	157.2[b]	13-cis, 15-anti	b
	L165	15.5[b]		166.0[b]	13-cis, 15-anti	b
	L174		123.3[b]	173.8[b]	13cis, 15-anti	b
	L181	24.0[b]	116.2[b]	181.2[b]	13-cis, 15-anti	b
	L185	24.5[b]	116.2[b]	186[b]	13-cis, 15-anti	b
	M_0	21.5[b]	124.5[b]<br>125.0[c]<br>123.2[c]	317[b]<br>296.4[c]	13-cis, 15-anti	b<br>c
	M_n		124.6[c]	288.8[c]	13-cis, 15-anti	c
	M			295.2[d]	13-cis, 15-anti	d
	N		115.2[d]	150.7[d]	13-cis, 15-syn	d
Y185F	AT	13.2[e]	123.1[e]		all-trans	e
	CS	21.7[e]	110.0[e]		13-cis, 15-syn	e
	CS*	18.0[e]	115.3[e]		13-cis, 15-syn	e
	N	19.2[e]	125.4[e]		13-cis, 15-anti	e
	O	13.2[e]	123.1[e]		all-trans	e

a, b, c, d, e : literatures which report the chemical shift values. References are listed in the last page.

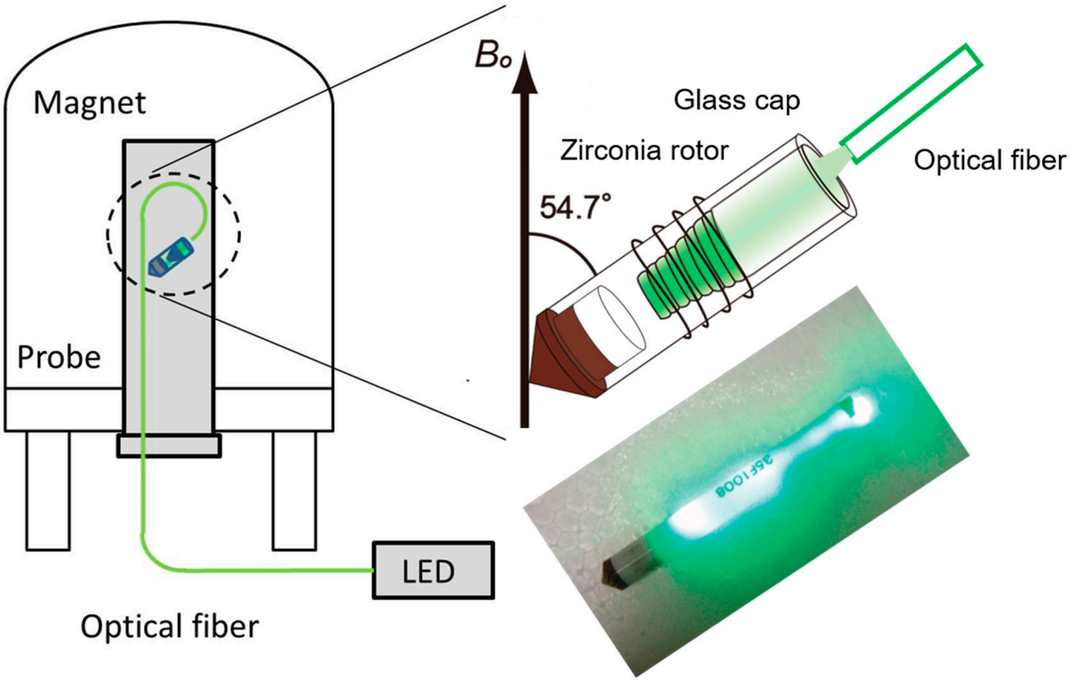

Figure S1. Schematic diagram of the *in situ* photoirradiation solid state NMR spectrometer. LED light sources (520 nm, 100 mW and 365 nm, 150 mW) are connected to optical fiber and guided from the bottom to the top part of the zirconia rotor in the probe head. The top part of the zirconia rotor is capped by a glass rod. Tip part of glass rod is ground to provide light illumination perpendicular to the rotor axis, so that light can be illuminated efficiently from inside the MAS rotor.

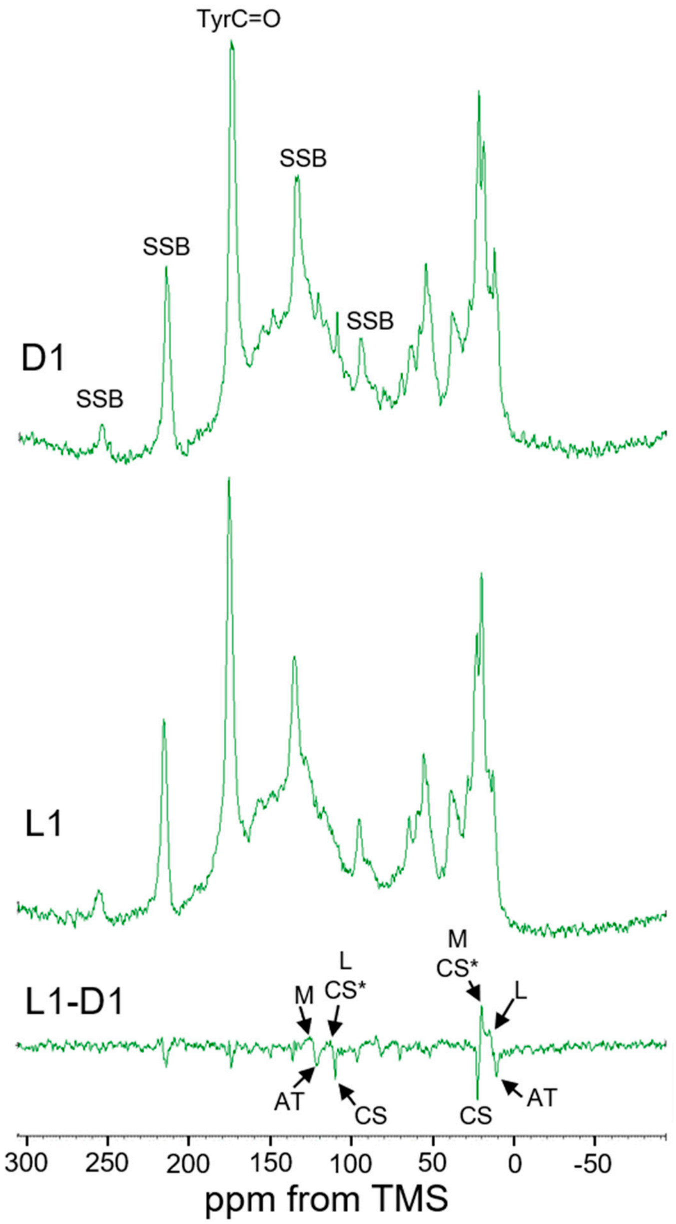

Figure S2. Full scale ^{13}C CP-MAS NMR spectra of [Tyr^{13}CO] and [20-^{13}C, 14-^{13}C]retinal-D96N-BR were recorded in the dark (D1; dark adapted state) and then under green light (L1) illumination at −30 °C. The difference in spectra between L1 and D1 (L1-D1) indicated that the AT state changed to the M and L intermediates and the CS state changed to the CS* intermediate. Line broadening factor of 60 Hz was applied. Scaling factor was not applied to get deference free induction decay signal between L1 and D1 that was Fourier transformed to get difference spectrum (L1-D1). [Tyr^{13}CO] and [20-^{13}C, 14-^{13}C]retinal-D96N-BR sample was used for light illumination experiments. However, only [20-^{13}C, 14-^{13}C]retinal-D96N-BR sample is described in the manuscript because NMR signals of [20-^{13}C, 14-^{13}C]retinal is mainly focussd on this study.

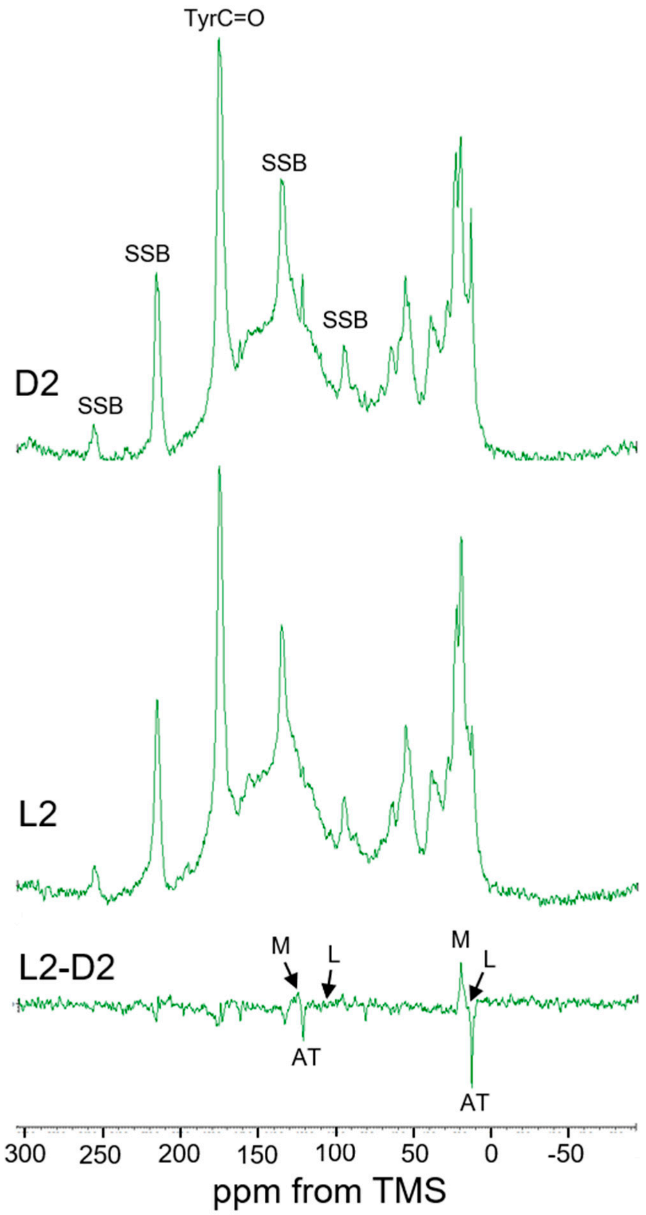

Figure S3. Full scale ^{13}C CP-MAS NMR spectra of [Tyr^{13}CO] and [20-^{13}C, 14-^{13}C]retinal-D96N-BR were recorded in the dark (D2; light adapted state) and then under green light (L2) illumination at −30 °C. The difference in spectra between L2 and D2 (L2-D2) indicated that the AT state changed to the M and L intermediates. Line broadening factor of 60 Hz was applied. Scaling factor was not applied to get deference free induction decay signal between L2 and D2 that was Fourier transformed to get difference spectrum (L2-D2). [Tyr^{13}CO] and [20-^{13}C, 14-^{13}C]retinal-D96N-BR sample was used for light illumination experiments. However, only [20-^{13}C, 14-^{13}C]retinal-D96N-BR sample is described in the manuscript because NMR signals of [20-^{13}C, 14-^{13}C]retinal is mainly focussd on this study.

References

a S. O. Smith, H. J. M. de Groot, R. Gebhard, J. M. L. Courtin, J. Lugtenburg, J. Herzfeld, R.G. Griffin, Structure and protein environment of the retinal chromophore in light- and dark-adapted bacteriorhodopsin studied by solid-state NMR. Biochemistry **1989**, 28, 8897-8904.

b V. S. Bajaj, M. L. Mak-Jurkauskas, M. Belenky, J. Herzfeld, R. G. Griffin, Functional and shunt states of bacteriorhodopsin resolved by 250 GHz dynamic nuclear polarization-enhanced solid-state NMR. Proc. Natl. Acad. Sci. USA, **2009**, 106, 9244-9249.

c J. G. Hu, B. Q. Sun, M. Bizounok, M. E. Hatcher, J. C. Lansing, J. Raap, P.J.E. Verdegem, J. Lugtenburg, R. G. Griffin, J. Herzfeld. Early and late M intermediates in the bacteriorhodopsin photocycle: A solid-state NMR study. Biochemistry, **1998**, 37, 8088-8096.

d K. V. Lakshmi, M. R. Farrar, J. Raap, J. Lugtenburg, R. G. Griffin, J. Herzfeld, Solid state ^{13}C and ^{15}N NMR investigations of the N intermediate of bacteriorhodopsin. Biochemistry, **1994**, 33, 8853-8857.

e K. Oshima, A. Shigeta, Y. Makino, I. Kawamura, T. Okitsu, A. Wada, S. Tuzi, T. Iwasa, A. Naito, Characterization of photo-intermediates in the photo-reaction pathways of a bacteriorhodopsin Y185F mutant using *in situ* photo-irradiation solid-state NMR spectroscopy, Photochem. Photobiol. Sci. **2015**, 14, 1694-1702.
